# Early implementation of the structured medication review in England: a qualitative study

**DOI:** 10.3399/BJGP.2022.0014

**Published:** 2022-06-28

**Authors:** Mary Madden, Thomas Mills, Karl Atkin, Duncan Stewart, Jim McCambridge

**Affiliations:** Department of Health Sciences, University of York, York.; PHIRST London, London South Bank University, London.; Department of Sociology, University of York, York.; Centre for Primary Health and Social Care, School of Social Professions, London Metropolitan University, London.; Department of Health Sciences, University of York, York.

**Keywords:** consultation standards, COVID-19, implementation, medication review, polypharmacy, primary health care

## Abstract

**Background:**

NHS England has introduced a new structured medication review (SMR) service within primary care networks (PCNs) forming during the COVID-19 pandemic. Policy drivers are addressing problematic polypharmacy, reducing avoidable hospitalisations, and delivering better value from medicines spending. This study explores early implementation of the SMR from the perspective of the primary care clinical pharmacist workforce.

**Aim:**

To identify factors affecting the early implementation of the SMR service.

**Design and setting:**

Qualitative interview study in general practice between September 2020 and June 2021.

**Method:**

Two semi-structured interviews were carried out with each of 10 newly appointed pharmacists (20 in total) in 10 PCNs in Northern England; and one interview was carried out with 10 pharmacists already established in GP practices in 10 other PCNs across England. Audiorecordings were transcribed verbatim and a modified framework method supported a constructionist thematic analysis.

**Results:**

SMRs were not yet a PCN priority and SMR implementation was largely delegated to individual pharmacists; those already in general practice appearing to be more ready for implementation. New pharmacists were on the primary care education pathway and drew on pre-existing practice frames, habits, and heuristics. Those lacking patient-facing expertise sought template-driven, institution-centred practice. Consequently, SMR practices reverted to prior medication review practices, compromising the distinct purposes of the new service.

**Conclusion:**

Early SMR implementation did not match the vision for patients presented in policy of an invited, holistic, shared decision-making opportunity offered by well-trained pharmacists. There is an important opportunity cost of SMR implementation without prior adequate skills development, testing, and refining.

## INTRODUCTION

Implementation is defined as any activity undertaken between making a commitment to adopt an innovation and the time when this becomes organisational routine, is no longer regarded as new, or is abandoned.^1^ NHS England has introduced a new structured medication review (SMR) service within primary care networks (PCNs) forming during the COVID-19 pandemic.^2^ The Additional Roles Reimbursement Scheme (ARRS) funded a PCN clinical pharmacy role and the Directed Enhanced Service (DES) contract made the SMR a PCN service requirement.^3^ Policy drivers were addressing problematic polypharmacy, reducing avoidable hospitalisations, and delivering better value from medicines spending.^4^ Polypharmacy has been identified as a ‘wicked’ problem of increasing scale and complexity for policy, with a limited evidence base on how to meet the challenge; that is, to support patients and healthcare professionals in the complex decision making involved.^5^

The SMR service is intended to be a patient-centred, outcome-focused approach to medicines optimisation,^6^ which will improve the quality of prescribing and reduce the risk of harm to patients, thereby alleviating workload pressures on GPs and delivering improvements to patient care and outcomes.^7^ The SMR specification in the DES contract is for an invited, personalised, holistic review of all medicines and their benefits to health for people at risk of harm or medicine-related problems, lasting ≥30 minutes.^7^ Consultations, conducted by pharmacists who have, or are in training for, a prescribing qualification and have advanced assessment and history-taking skills, are intended to be attentive to health literacy and conducted in line with the principles of shared decision making.^7^ Expert peer guidance recommends allowing additional time for preparation.^8^ A subsequent Department of Health and Social Care Medicines Directorate report on medicines optimisation identified the SMR as *‘an ideal tool to help people with problematic polypharmacy’*.^9^ The Secretary of State for Health and Social Care has accepted the report’s recommendation that NHS England and NHS Improvement should expand the use of SMRs in PCNs, with appointments lasting at least 30 minutes to allow for shared decision making, and with social prescribing link workers trained to help support patients after an SMR.^9^ Guidelines on shared decision making were published by the National Institute for Health and Care Excellence (NICE).^10^

The emphasis on time, personalisation, and patient outcomes in SMR specifications stem from concerns about quality, consistency of approach, and selectivity of patients in the now decommissioned Medicines Use Review (MUR) service in community pharmacy.^11^^–^13 After speedy implementation, without appropriate feasibility testing and refinement, problems were identified in training, service introduction, and service targeting, which led to variability in delivery of the MUR.^14^^,^^15^ Owing to the COVID-19 pandemic, PCNs are still at a relatively early stage in the process of SMR implementation.^2^ The introduction of the service was delayed, with the number of SMRs to be determined by clinical pharmacist capacity.^7^ Reporting from an ongoing longitudinal study, this article explores early implementation from the point of view of the primary care clinical pharmacist workforce. It provides insights into how the SMR innovation was being interpreted and implemented on the ground when first introduced, including how SMRs were being distinguished from other forms of medication review.^16^

**Table 1:** How this fits in

The structured medication review (SMR) service is a new patient-centred, outcome-focused approach to medicines optimisation implemented by pharmacists working in primary care networks (PCNs). Its introduction has been protracted owing to the COVID-19 pandemic. The SMR is intended to alleviate workload pressures on GPs and deliver improvements to patient care and outcomes, particularly problematic polypharmacy. This study found that the SMR is currently not well organised in clinics; arrangements for patients vary considerably, it is often provided as an *ad hoc* service by telephone, and implementation of the intended service is patchy. Departures from SMR service specifications could have important short-and long-term implications for patient-centred practices; further study of implementation is required if the service is to meet policy aspirations.

## METHOD

Two semi-structured interviews were carried out with each of 10 newly appointed pharmacists (20 interviews in total) in 10 PCNs in Northern England between September 2020 and June 2021. In addition, 10 pharmacists in 10 other PCNs across England already established in GP practices, were interviewed once between February and May 2021. Interviews were conducted via video call by one of two researchers using a semi-structured topic guide (see Supplementary Box S1). Audiorecordings were professionally transcribed and pseudonymised. A modified framework method was used to organise and present data from transcripts.^17^ This supported a constructionist thematic analysis.^18^ With the topic guide forming the initial framework, interview transcripts were coded in NVivo (version 12) to produce a list of initial descriptive themes identifying SMR understanding and practices. Comparative analysis identified common, recurring, and conflicting perspectives, and noted the ways in which accounts were constructed. Preliminary analysis of sample scripts, subthemes, and the final analytic narrative were discussed with co-investigators. The study forms part of a research programme on the inclusion of alcohol within SMRs delivered by clinical pharmacists working in PCNs.^19^

All of the pharmacists conducting SMRs in this study’s sample did so remotely by telephone. Three of the 10 newly employed ARRS pharmacists were appointed at senior/lead pharmacist level; two of these had been qualified for 4 years and one for 30 years. One was provisionally registered. Eight had applied for the PCN position from community pharmacy, one from hospital pharmacy, and one from a GP practice pharmacist position. Of the eight from community pharmacy, the pharmacist with 30 years’ experience had also worked in industry and at commissioning level, two others had some pre-registration experience in hospital, and one had worked in a private clinical services company. All 10 newly employed ARRS pharmacists were undertaking or had just completed the compulsory 18-month primary care pharmacy education pathway (PCPEP) run by the Centre for Pharmacy Postgraduate Education (CPPE).^20^ Two were prescribers. Some were working within one GP practice, while others split their time across PCN practices. Most had pharmacist colleagues but others were the first and sole pharmacist in the PCN.

All of the 10 established GP practice pharmacists were prescribers and most were in or about to take on senior and leadership roles in PCNs and integrated care services. They had completed the Clinical Pharmacists in General Practice: Pilot Scheme (GPPTP), launched in 2016–2017.^21^ Five had previously worked in hospital pharmacy and three at commissioning level. Further participant characteristics are available in Supplementary Table S1.

## RESULTS

### Classifying reviews and calibrating competence

Experienced GP practice and senior pharmacists said that SMRs took more time and were more challenging to do than other medication reviews because they were more clinically complex, in-depth, and patient focused. Some likened them to the reviews they already did on frailty and in care homes, others to current level two ‘treatment reviews’ with a patient present and more time allowed. All reviews undertaken in GP practices were compared favourably against MURs in community pharmacy, and those more experienced in clinical reviews said that it took time to develop the necessary knowledge and skills:

*‘Honestly, it’s* [MUR] *a waste of time really and I think it’s a good idea they scrapped it, it was purely just a tick box exercise … to get the money … it was a … level one review … how are you getting on with your medicines, do you take them all the time? … Whereas … I try and teach my pharmacists … to do a proper medicine review, it’s a treatment review, reviewing their bloods, the indication … is it working for them and even if it’s all good, do they need to be on it still? … it’s far, far more in-depth.’*

(ARRS pharmacist [A]9, second interview [2])

A lead pharmacist with 13 years’ experience in GP practice, who continued to work half time in community pharmacy, emphasised the difference, and explained their perception of the accompanying lack of confidence those coming in from community pharmacy could feel:

*‘I have … two pharmacists that have come from community, to line manage, ‘cause I know their experience. And I still work in community, so I know exactly how they’re feeling … there’s not a lot of appreciation … it’s a big change confidence-wise … if you’re in one of those 10 000 plus* [item] *, check, check, check, check, check* [pharmacies] *, your clinical knowledge is rubbish after years … even when you clinically check a script, you’re looking … at what’s been prescribed, not the patient as a whole … when you’re in a surgery, you’re looking at everything … that clinical knowledge has to be there … it’s not necessarily that you’re not equipped to do it …* [but] *you’ve* [had] *a constant thing in the back of your head saying there’s a script sitting there waiting. Someone’s going to come back in two minutes … ’*

(GPPTP1)

A senior GP practice pharmacist noted confusion caused by the association of the SMR with the ARRS role, and therefore a workforce potentially less experienced in clinical medication reviews:

*‘… all of our reviews were probably that holistic review … so I would say … we were doing them before with a different name …* [we] *felt at first that we weren’t going to able to do them, it was mainly for the new PCN roles … I think that’s been clarified since … but it’s taken quite a while for that to … filter through. And … what do we still class as a medication review, what do we class as an SMR? … it feels very unfair to put a new pharmacist who’s only just started on a pathway in with all the complex … you don’t know what you don’t know until you’ve come across it, so it’s been a bit of a tricky one* . *’*

(GPPTP2)

An ARRS pharmacist who had moved from community pharmacy to a PCN where she was the sole pharmacist, before moving on to another PCN to take up a leadership role, said it had taken some time to realise the difference between levels of review in primary care:

*‘… when I actually moved into primary care, I didn’t really know where the heck to start and as much as I’ve done medication reviews … in community, they all felt very simplified compared to what I’m currently doing … I don’t think a lot of people fully understand the process of doing a structured medication review … when I started in my PCN … some pharmacists were doing big medication reviews and … coding them as SMRs … when I actually went into what the SMR involved, they are definitely not doing SMRs … what they … were doing was more … like resolving a query … I think the quality of my SMRs now is massively different from when I first started. And I think I’ve just undergone a massive transformation … But I think with more knowledge, you … almost … become consciously incompetent because you realise what you don’t know* . *’*

(A4:2)

Other ARRS pharmacists attenuated differences between reviews, other than targeting specific patient groups and, in the case of the MUR, access to clinical records.

ARRS pharmacists discussed shared decision making in SMRs largely in the form of not making changes without letting patients know, and also as a positive action, securing patient agreement and compliance through information-giving:

*‘… shared decision making is kind of like the pivotal backbone of a consultation because without that communication and decision making from the patient side … how do we know they’re going to comply?’*

(A7, first interview [1])

This pharmacist also gave an example of not having a sense of how her consultation practice was developing. A colleague overheard her phone call:

*‘I thought I was doing a really good consultation … and doing shared decision making. I put the phone down. One of the pharmacists she said, oh no, you sounded a bit harsh … I thought … I worded it really well … And only when that pharmacist said that did, I think, “Oh what if they’re thinking that?”’*

(A7:1)

ARRS pharmacists from community pharmacy backgrounds were particularly concerned to improve clinical knowledge:

*‘Before, I was scared to speak to the patients about their medical condition because I wasn’t competent enough with the medical stuff … I was lost and I was scared to ask if the patient has any sort of heart failure problem and if they have, what should I do now? So let’s avoid those questions* . *’*

(A1:2)

ARRS pharmacists said a hospital background helped with clinical knowledge, but this was largely on medicines reconciliation and appropriateness, with limited patient interaction. A senior ARRS pharmacist who had worked mostly in general practice said the profession lacked the hands-on, face-to-face training with patients that doctors and dentists receive. They criticised pharmacy training for encouraging pharmacists to interpret guidelines as rules, meaning they were ill equipped for the ambiguities in primary care practice that underpin shared decision making:

*‘… Pharmacy school is, right or wrong … it’s almost like the guideline is the law and NICE is the law … whereas the GPs don’t have that view … I think it makes pharmacists feel uncomfortable, the lack of certainty around it is difficult* . *’*

(A9:1)

### ‘Letter versus spirit’ of the new SMR service model

There was uncertainty about what to do with the autonomy afforded to PCNs in the identification and prioritisation of patients for SMR within the DES criteria. Some pharmacists (more senior or working alone) were actively running searches of patients taking multiple medications and, given the large numbers produced, were discussing how to refine targeting at practice or PCN level. Others said searches were or would be run at the level of practice, PCN, or clinical commissioning group. Rather than an appointment-based, invited service, SMRs outside care homes were mostly being offered reactively on an *ad hoc* basis, and determined by individual pharmacists during routine medication reviews:

*‘… it’s on me. I decide who to review and … it’s a fine line, where does a medicine review stop and a structured medication review start? …* [T] *here’s the criteria … polypharmacy … medicines more prone to mistakes … I’ve got the list written somewhere* . *’*

(A3:2)

Routine medication reviews could be re-classified as SMRs if the patient fitted ≥1 of the DES criteria. In addition, some were receiving SMR referrals from GPs where patients required a more in-depth review. *Ad hoc* SMRs were also considered ‘filler work’ in between other tasks. All pharmacists, although pleased that there were no targets set for SMRs during the pandemic, anticipated that these would soon be introduced. Citing the MUR as an example, most expressed concerns that quantity might displace quality of practice, combined with an awareness that achieving targets was a key criterion for determining success. Some ARRS staff, therefore, attempted to maximise the number of SMRs achieved on the basis that targets appeared inevitable:

*‘I always felt that … I’m just going to get an email … saying, right, you should be doing x number per week now. So … it’s best to get started … so it’s not a shock … plus, even though there’s no target, someone could still look back and say, why didn’t you do any in this week? … So … I tried to get started on them as soon as the DES was released … I was going to have to do them eventually anyway, I may as well get used to them … any* [reviews] *that I get sent, if I can figure out a way to wangle them as an SMR I will do. And … when I’ve got time to kill, and I seek out patients, I will generally seek out the ones who are eligible for SMRs first.’*

(A3:2)

Some of the GPPTP cohort were frustrated about a lack of support for SMRs at GP practice level, and valuing the service, were trying to find ways of showing its value to GP colleagues without additional time cost by integrating it into usual practice:

*‘… quite frankly, the practice couldn’t give a stuff what I do it on, as long as it gets done and I can do the other things that they want me to do … I think the GPs think it’s a bit of pain really … ’*

(GPPTP7)

Pharmacists booked to do a medication review telephoned the patient during that time:

*‘I’ve got one booked today … I don’t think they know* [to expect the call] *… if someone doesn’t pick up … we’ll text them and we’ll say, “I’m ringing you from a no reply number, I’ll try again later”. We ring them back three times, that’s our maximum. If we don’t get them that day, we’ll rebook them for another day* . *’*

(A7:2)

This ‘cold calling’ was perceived as not ideal, but practical. An ARRS pharmacist who knew that patients were to be invited to SMRs to explain the service and allow preparation described it in terms of the ‘letter versus the spirit of the law’:

*‘… the spirit of the law is that they be invited in plenty of time and they can prepare any questions they’ve got, they can bring their meds with them. But, the letter of the law, as the guidance is written, I think would still allow cold calls … ’*

(A3:2)

SMRs booked as standard medication reviews had to fit into the time allowed. Time for a longer consultation could be found if other reviews were short:

*‘… it tends to be … kind of,* ad hoc *… somebody will appear on my telephone list and it’ll just say, medication review and one patient will have, like, three items and the next one will have 33 and you’re still given your 10-minute slot and it’s tricky … a lot of them are just, kind of, trying to work out exactly what the issue is* . *’*

(GPPTP3)

Rather than turning one medication review service into another, one of the more experienced ARRS pharmacists who specialised in clinics for chronic obstructive pulmonary disease (COPD) invited those she identified as eligible for an SMR to a separate appointment for which they could prepare:

*‘I never cold call … if somebody expects the phone call, they’re better prepared … I’ve done it before with pain management and … the reductions of opioids. And as soon as you just phone somebody randomly and say, “Hi it’s … the pharmacist from … I’m trying to call today we’re going to have a look at your pain medications”. Straight away you’ve got the brick wall. I think, if somebody is prepared and knows, then you get a lot more out of that consultation* . *’*

(A2:2)

While recognising invitations and appointments as a practice ideal, there was doubt about the time and capacity to implement these as described in the DES:

*‘… so we’re fully aware that we should be formally inviting people in. We’re not, we’re just ringing them … they’re an elderly population generally, so you’re less able to text them and email them* [and] *the practice doesn’t want to be sending a lot of letters because they don’t like postage costs … for that older group, it’s quite a challenge of how you effectively communicate with them without confusing them. And sometimes they just need someone to talk to and so it’s easier just to ring them up … at the moment we’re struggling in terms of space and … we don’t even have a phone each, so it’s difficult* . *’*

(GPPTP2)

This pharmacist shifted from explanation based on resource limitations to justification based on her view of the needs of older people. The focus generally was on how to fit patients into the existing structures. Suggestions to better organise SMRs were to invite patients to see the pharmacist when coming in for something else and to get pharmacist technicians to do the preparatory work.

### Consultation length and structure

For *ad hoc* SMRs, the suggested 30 minutes was interpreted to include preparation and writing up time, with 15–20 minutes given to the actual consultation. A senior ARRS pharmacist with a background in GP practices said that while his preparation time had reduced with experience, it took some time before he did reviews well:

*‘…* [it] *took a long time to get good at it … At the beginning, I used to sit there for 10 minutes before doing one, write all of my notes down of what I could probably ask the patient and where I want to go with it and then I’d call them, and look at my notes … you realise, actually, that there’s so much to learn, because if you want to do a proper full review of the medicine, you need to know about the condition … a big learning curve, because, at the beginning … I didn’t feel competent to do such a detailed review and, over time, you know, I’ve got better* . *’*

(A9:2)

An ARRS pharmacist yet to do an SMR was concerned about how she would deal with multiple issues in one consultation and, rather than valuing the longer, holistic SMR service model addressing all medications, expressed a preference for a series of shorter, separate condition-focused or medication-focused consultations:

*‘… so if you had somebody that had COPD and heart failure, what else, was also a diabetic, they might have osteoporosis. And there’s issues with every single one of those, you’re never going to get that done in half an hour … And I think the patient wouldn’t know whether they were coming or going with it if you tried to tackle all of that at once either. So … I think it’s better done as three 10-minute appointments rather than a half hour one … it’s too much for me all at once, never mind the person on the other end of the phone* . *’*

(A5:2)

Most were unclear what the ‘structured’ in the SMR referred to. Some ARRS pharmacists were looking for a template to help structure the consultation and regarded the template used to record SMRs on their clinical systems as, *‘basically a guide of how to do one’* (A8:1), although unwieldy to use:

*‘… it was all over the place … At one point it would be asking you about compliance, then it’ll be asking what’s the medicine for, then it’s asking about alcohol, and I just thought, “I need to take the bits which I think look relevant”* . *’*

(A8:1)

Those with longer experience regarded templates as tools for data capture rather than consultation structure:

*‘... I think prompts are okay maybe but I think a lot of people seem to want a template of “this is how you do an SMR” … the whole point is that they should be holistic … they should be focusing on what that patient … their… issue is and that’s going to be different for everybody … I guess that’s just something that comes with time and training … ’*

(GPPTP2)

This sense that some were slavishly following templates that impeded the flow of a consultation was described by other experienced pharmacists:

*‘I think the problem is it’s called a structured medication review and therefore I expect that people think right, I’ve got to conform to the structure and that’s not the case at all. I think it’s been wrongly named … because there really isn’t a structure as such* . *’*

(A4:2)

## DISCUSSION

### Summary

New to working in GP practices, ARRS pharmacists drew on what they learnt on the PCPEP, but also from the frames, habits, and heuristics with which they were already familiar, including the target-driven incentives of corporate community pharmacy. This shifted SMR focus onto pharmacist activity (productivity) rather than outcomes for patients. Those lacking patient-facing expertise sought template driven, institution-centred practice.

SMR implementation was largely delegated down to individual pharmacists, and adaptation to the complexities of forming PCN systems risked undermining the purposes of the service. The new clinical pharmacist workforce was reactive to the needs of individual GP practices facing ongoing workforce and pandemic pressures. Early SMR implementation did not, therefore, match the ideal for patients presented in policy documents of an invited, holistic, shared decision-making opportunity offered by prescribing pharmacists experienced in history taking. There is an opportunity cost of SMR implementation without prior adequate skills development, testing, and refining, as with the MUR.^14^ Conforming to the perceived realities of pressurised general practices may be setting unhelpful precedents for future SMR conduct.

### Strengths and limitations

This study adds to understanding of the implementation of the new SMR service and, relatedly, includes data on pharmacists’ perceptions of their medicines review roles, competencies, and practice environments derived from detailed, in-depth interviews. The latter content on skills development, SMR conduct, and the PCN and ARRS role context requires dedicated attention and will be reported elsewhere. The study has limitations common to exploratory qualitative studies. Comparison with observation of SMR practice is needed to ground the findings in the empirical realities of practice. Following a cohort over a longer period will provide insight into how the SMR service develops and how pharmacists’ perceptions of their consultation confidence and competence develop over time, as they complete the PCPEP, become prescribers, and gain experience of clinical assessment and history taking.

### Comparison with existing literature

Implementation science identifies the importance of understanding the ‘fit’ between an intervention and the context in which it is being implemented, to understand how change is achieved and identify the causes of evidence to practice gaps.^22^^,^^23^ Both knowledge about multiple conditions and communication skills are important preparation for SMRs. If pharmacists are to have the confidence to address the complexity of patients’ clinical and social situations, discuss the balance of different potential harms, and know when and how to raise possibilities for de-prescribing or changing prescriptions, then extended support of the acquisition and maintenance of clinical and communication skills are required. An evaluation of the preceding GPPTP scheme found newly recruited pharmacists recognised gaps in their knowledge and skills when performing an extended role in general practice, but were not always able to identify their own specific learning needs.^21^ Despite developments made to the subsequent PCPEP, the findings indicate ongoing deficiencies in grounded skills development for patient-facing medication reviews. Guidance on consultation practice may make more sense to the more experienced *‘consciously incompetent’* pharmacist (A4:2) becoming familiar with clinical uncertainty and complexity, than to newer appointees preoccupied with initial gaps in their clinical knowledge. Knowledge and skills issues need to be disaggregated in appreciation of practice development needs, with careful attention given to the contexts in which skills are developed and honed; that is, the everyday institutional practices that underpin problematic polypharmacy and wider service-level responses to it.^5^

### Implications for research and practice

At this early stage of implementation, SMR practice outside of care homes appears to have largely developed by fulfilling a variety of routine medicines-related tasks in response to backlogs. Variations in quality and outcomes, and limited progress in developing more patient-centred approaches in medication reviews, are to be expected in these circumstances. This study shows there is more to be done on the ground to distinguish between different types of review and how they are organised, and to clarify their specific purposes and fit for a particular patient’s care.^24^ Departures from SMR service specifications could have important short-term and long-term implications for patient-centred practices, which need to be better understood. If the service is to meet policy aspirations, therefore, it is important to study progress in the implementation of the SMR as a new service distinct from other kinds of medication reviews from the perspectives of both practitioners and patients. Studies of how SMRs are being implemented within multidisciplinary teams and the involvement of other healthcare professionals are needed. The ongoing implementation of the SMR service thus requires much further study.

In practice, it needs to be recognised that clinical pharmacists and SMRs offer important new resources for addressing patient needs and population health issues. Not least, a 30-minute invited appointment at which multiple issues can be discussed. This has potential value; however, given the limitations of existing evidence, careful attention to strategic decision making about resource use is needed. GPs implementing pharmacist-run SMRs should clarify the distinct purposes of the SMR for their particular practice population and recognise that, because pharmacists have less in-depth experience of consulting with patients, they will require additional supervision to develop the required history-taking and shared decision-making skills. Newer ARRS pharmacists in particular may benefit from more carefully supported introductions into patient-centred SMR practice, aided by clinically experienced pharmacists, to help build confidence and competence.
